# Ellagic acid Alleviates hepatic ischemia–reperfusion injury in C57 mice via the Caspase-1-GSDMD pathway

**DOI:** 10.1186/s12917-022-03326-0

**Published:** 2022-06-18

**Authors:** Hao Wang, Fujun Miao, Delu Ning, Chunlan Shan

**Affiliations:** 1 College of Animal Science, Guizhou University, Guiyang, 550000 China; 2 College of Veterinary Medicine, Yunnan Agricultural University, Kunming, 650201 China; 3 Yunnan Academy of Forestry and Grassland, Kunming, 650204 China

**Keywords:** Ellagic acid, Hepatic IRI, Pyroptosis, Caspase-1-GSDMD pathway

## Abstract

**Background:**

Ellagic acid (EA) has improving function against oxidative damage and inflammatory reaction in many disorders. Hepatic ischemia–reperfusion injury (IRI) is a common pathophysiological phenomenon in the veterinary clinic. In the present study, the protective effects of EA pretreatment against hepatic IRI-induced injury and the underlying mechanisms were investigated.

**Results:**

We found that pyroptosis is involved in hepatic IRI, which is manifested in increasing the expression of pyroptosis-related genes and promoting the expression of active caspase-1, thereby cleaving GSDMD-N to cause pyroptosis, and caspase-1^−/−^ mice were used to verify this conclusion. In addition, we found that EA protects against hepatic IRI by inhibiting pyroptosis, including reducing the activity of caspase-1 and its expression in the liver, inhibiting the lysis of GSDMD-N, and reducing the levels of IL-18 and IL-1β.

**Conclusions:**

The present results have demonstrated that prophylactic administration of EA ameliorated hepatic IRI by inhibiting pyroptosis induced in hepatic ischemia–reperfusion *in vivo* through the caspase-1-GSDMD axis, providing a potential therapeutic option prevent hepatic IRI in pets.

**Supplementary Information:**

The online version contains supplementary material available at 10.1186/s12917-022-03326-0.

## Background

Liver operations are common in veterinary surgery and the blood supply of the liver is interrupted. Restoration of the blood supply following ischaemia may lead to further liver damage, which is called hepatic ischemia–reperfusion injury (IRI) [1]. Multi-protein complexes called inflammasomes are related to the pathogenesis of hepatic IRI and are considered to be a key factor in liver cell damage [2]. The NLRP3 inflammasome is a protein complex located in cells, and is composed of NLRP3, ASC and pro-caspase-1.

Blood flow restoration triggers tissue inflammation and ischemic damage by activating inflammasomes [3]. After the inflammasome activation, the affected tissues undergo apoptosis and pyroptosis, another type of inflammation-related cell death [4, 5]. Pyroptosis is a form of lytic programmed cell death initiated by GSDMD-N [6, 7]. It is mainly mediated by the activation of inflammatory complexes such as NLRP3, and the activation of its downstream factor caspase-1, accompanied by an inflammatory response [5]. Although there is no direct evidence for the presence of pyroptosis in hepatic IRI, the activation of inflammasomes in hepatic IRI has been elucidated, indicating that pyroptosis is involved in hepatic IRI [8]. Interestingly, previous studies have shown that inhibiting pyroptosis can improve hepatic IRI and inhibit inflammation [9]. Before or during liver surgery, using drugs or small molecules to activate key survival pathways or inhibit pyroptosis pathways can help reduce hepatic IRI damage [10].

The term Ellagic acid (EA) comes from the French word acide ellagique, and its molecular formula is C_14_H_6_O_8_ (2,3,7,8-tetrahydroxy [1]-benzopyranol[5,4,3-cde]benzopyran-5,10-dione). EA is a naturally occurring polyphenolic compound that is found in many fruits, walnuts and plant extracts in the forms of hydrolyzable tannins called ellagitannins, such as strawberries, grapes, pomegranates and walnuts [11–14]. As reported in previous studies, EA possesses antibacterial, anti-inflammatory, pneumoprotective, nephroprotective, and cardioprotective properties [15]. Kang et al. reported that EA can scavenge free radicals and reduce the production of inflammatory factors such as IL-6, TNF-a and IL-1β [16].

Recent studies have shown that EA can reduce inflammation and exerts a protective effect on the liver [17]. The research from Elyamany M. shows that EA treatment can significantly reduce the liver damage caused by valproic acid in rats [18], and L. Gu et al. demonstrated that EA protected against LPS/GalN-induced liver injury in SD rats by enhancing the antioxidative defense system and reducing the inflammatory response [19]. Kim, D described a significant reduction in endotoxemia and inflammatory liver damage with EA treatment by inhibiting the imbalance of the intestinal flora in c57 mice, elevated oxidative stress and apoptosis marker proteins [20]. However, the effect and underlying specific molecular mechanisms of EA on hepatic IRI remain unclear. Thus, in order to investigate the role of pyroptosis in hepatic ischemia–reperfusion and the intervention of EA in this process, we conducted studies using wild-type and Caspase-1^−/−^ C57BL/6 mice. In this study, the role of pyroptosis in hepatic ischemia–reperfusion injury was evaluated, illustrating that EA inhibits pyroptosis through the caspase-1-GSDMD pathway, thereby protecting the liver from ischemia–reperfusion injury.

## Results

### Caspase-1-GSDMD-induced pyroptosis occurs in the mice hepatic IRI model

**Fig. 1:**
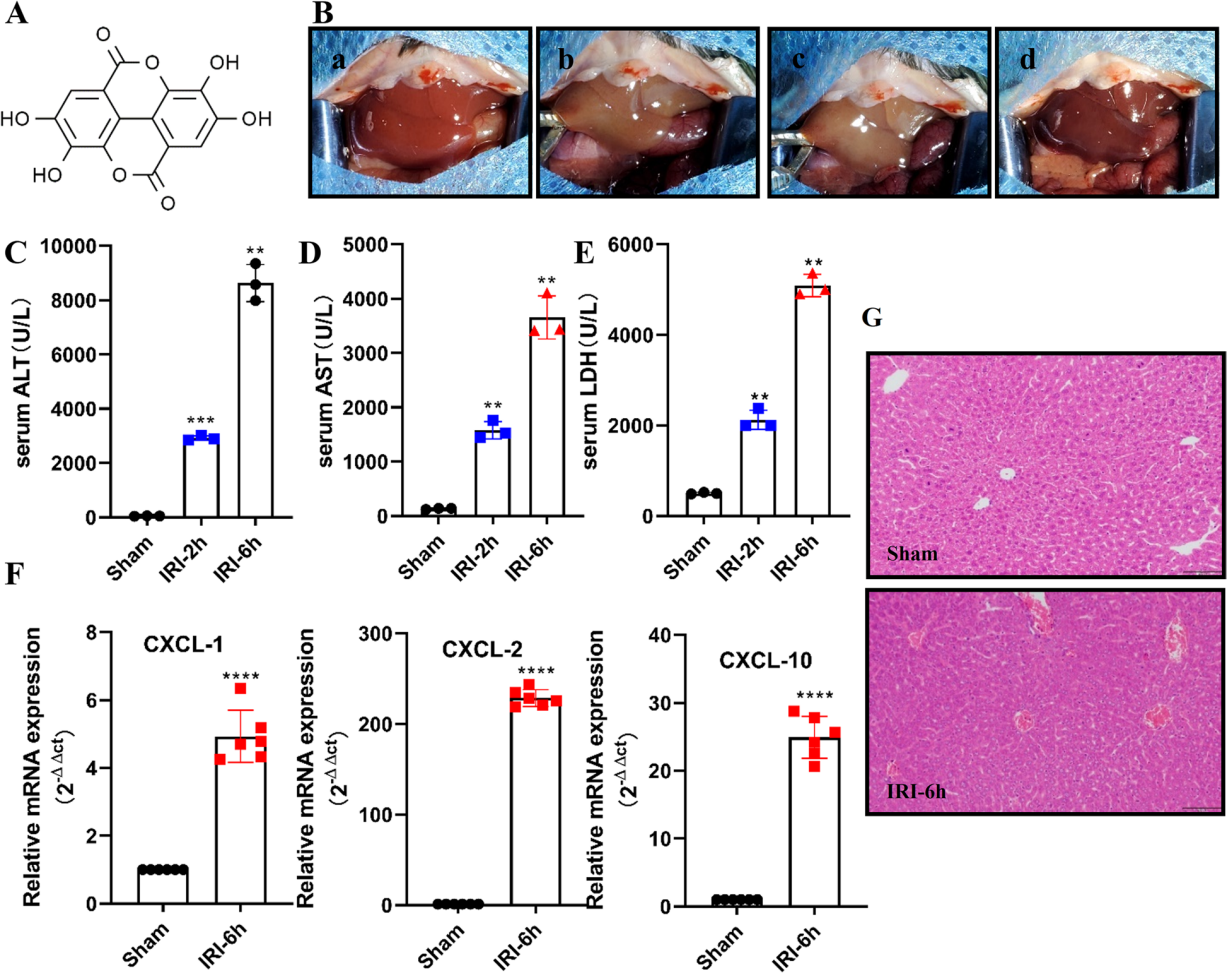
Severe liver damage caused by ischemia–reperfusion. **A** Chemical structure of ellagic acid. **B** C57/B6 mice were subjected to hepatic IRI model (a. before hepatic ischemia, b. start hepatic ischemia, c. ischemia for 90 min, d. reperfusion). **C**, **D** and **E** The hepatocellular function in serum samples was evaluated by detecting ALT, AST, and LDH levels (*n* = 3). **F** The relative expression levels of CXCL-1, CXCL-2 and CXCL-10 were determined using qRT-PCR in mice liver specimens (*n* = 6). **G** Representative images of hematoxylin and eosin (HE) staining (scale bar, 100 μm). All data are shown as the mean ± SEM.****P < 0.0001, **P < 0.01

The chemical structure of Ellagic acid was shown in Fig. 1A. A mice hepatic IRI model was established (Fig. 1B). The effects of the operation on mice livers subjected to 90 min of warm ischemia followed by 2 h and 6 h of reperfusion were analyzed. As illustrated by the serum ALT (Fig. 1C), AST (Fig. 1D) and LDH (Fig. 1E) levels, reperfusion for 6 h after ischemia caused more severe liver damage. The qPCR results showed that members of the CXC subfamily of chemokines, including CXCL-1, CXCL-2 and CXCL-10, were up-regulated in liver ischemia–reperfusion injury (Fig. 1F). As shown by histopathology (Fig. 1G), liver sections from mice undergoing hepatic IRI presented with significant features of severe centrilobular ballooning, congestion and lobular necrosis compared to those from sham-operated mice.

**Fig. 2:**
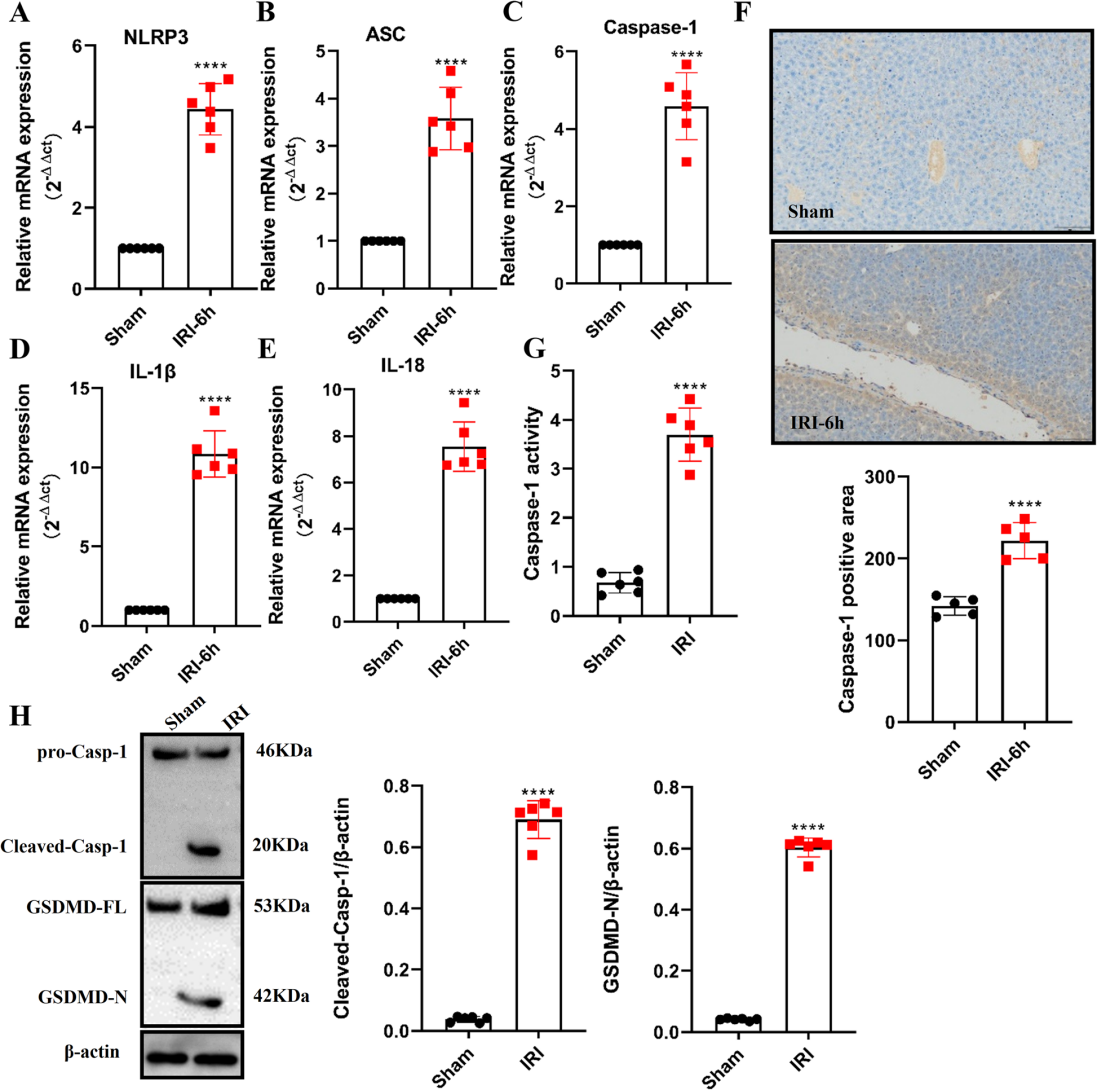
Caspase-1-GSDMD-induced pyroptosis occurs in the mice hepatic IRI model. **A**-**E** Relative expression levels of inflammasome-associated genes were determined using qRT-PCR in mice liver specimens (*n* = 6). **F** Representative images of caspase-1 staining (scale bar, 100 μm), the area of Caspase-1-positive cells in the livers were also determined (*n* = 6). **G** Caspase-1 activities were measured in mice hepatic IRI liver tissues (*n* = 6). **H** Examination of the proteolytic cleavage of Caspase-1 and GSDMD in mice liver specimens with Hepatic IRI or sham intervention, using immunoblotting analysis. GSDMD-FL, full-length GSDMD; GSDMD-N, the N-terminal cleavage product of GSDMD (*n* = 6). All data are shown as the mean ± SEM. ****P < 0.0001, ***P < 0.001

The pyroptosis induced by caspase-1-GSDMD could play a role in hepatic IRI. As shown in Fig. 2A to E, the expression of all inflammasome-associated genes in the liver tissues of the hepatic IRI mice model was assessed, with levels of NLRP3, ASC, Caspase-1, IL-1β and IL-18 transcripts being remarkably elevated in the hepatic IRI model mice, as compared with the sham group. Furthermore, the expression of caspase-1 increased in the liver of hepatic IRI model mice (Fig. 2F). Simultaneously, the serum levels of caspase-1 activity were up-regulated in hepatic IRI model mice (Fig. 2G). In addition, due to the cleavage of activated caspase-1 and the formation of the activated N-terminal domain of GSDMD (GSDMD-N), it has recently been identified as the executioner of pyroptosis [21, 22]. We next examined whether hepatic IRI could induce proteolytic cleavage of GSDMD. The results confirmed that significant degrees of both caspase-1 and GSDMD cleavage occurred in hepatic IRI (Fig. 2H). Conjointly, these data support the notion that Caspase-1-GSDMD-induced pyroptosis occurs in the mice hepatic IRI model.

### Depletion of Caspase-1 can reduce liver damage and inflammation caused by hepatic IRI

**Fig. 3:**
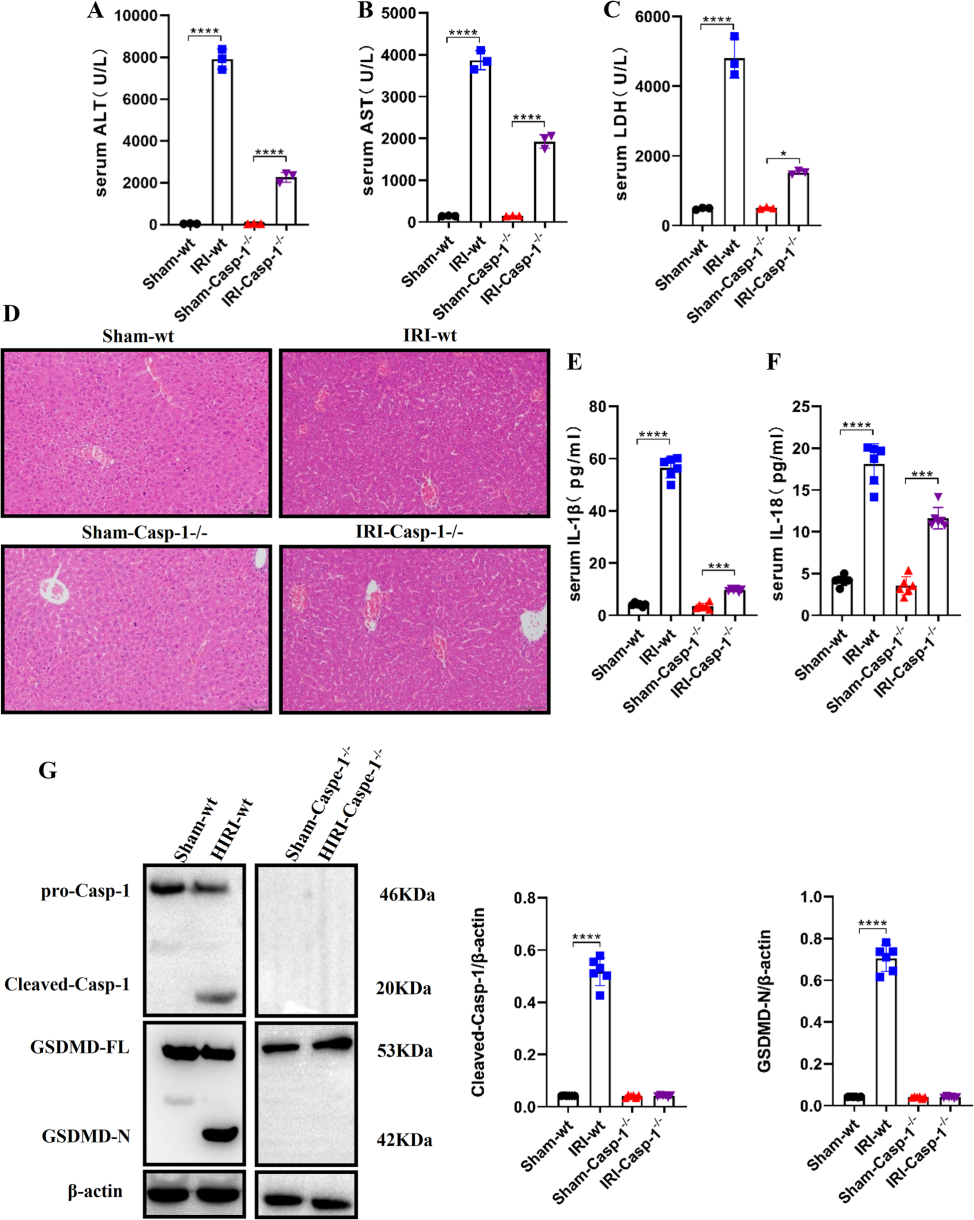
Depletion of Caspase-1 can reduce liver damage and inflammation caused by hepatic IRI. **A**, **B** and **C** The hepatocellular function in serum samples was evaluated by detecting ALT, AST, and LDH levels (*n* = 3). **D** Representative images of hematoxylin and eosin (HE) staining (scale bar, 100 μm). **E** and **F** IL-1β and IL-18 were measured with ELISA in liver samples of WT and Caspase1^−/−^ mice with Hepatic IRI and sham intervention (*n* = 6). **G** Examination of the proteolytic cleavage of Caspase-1 and GSDMD in mice liver specimens with Hepatic IRI or sham, using immunoblotting analysis. GSDMD-FL, full-length GSDMD; GSDMD-N, the N-terminal cleavage product of GSDMD (*n* = 6). All data are shown as the mean ± SEM. ****P < 0.0001, ***P < 0.001, **P < 0.01 and *P < 0.05

Based on the findings that caspase-1-GSDMD mediated pyroptosis could play a role in hepatic IRI, caspase-1–null background mice were used to further evaluate the role of pyroptosis in the pathogenesis of hepatic IRI. Specifically, the hepatic IRI or sham operation was performed on WT or *Caspase-1*–deficient (Caspase-1^−/−^) mice. WT mice displayed higher ALT, AST, and LDH levels in the hepatic IRI model as compared with sham groups. In contrast, the ALT, AST, and LDH levels of hepatic IRI Caspase-1^−/−^ mice were similar to those of the sham groups at 6 h of reperfusion (Fig. 3A, B, C). The histopathological examination further indicated that Caspase-1^−/−^ mice were spared from ischemia reperfusion-induced hepatic damage, as opposed to the hepatic IRI WT mice (Fig. 3D). Moreover, when the expression levels of proinflammatory cytokines IL-1β and IL-18 were measured in liver tissues, the depletion of caspase-1 abolished the increase of IL-1β and IL-18 of hepatic IRI model mice, as compared with their WT counterparts (Fig. 3 E and F). In addition, our results also showed that the hepatic IRI model triggered cleavage of GSDMD, while caspase-1 ablation attenuated GSDMD cleavage, as assessed by immunoblotting analysis. This indicates the inhibition of pyroptosis in the mice livers (Fig. 3G). Accordingly, the liver damage and inflammation caused by hepatic IRI could be restrained by limiting Caspase-1–mediated pyroptosis.

### Ellagic acid reduces liver injury in hepatic ischemia–reperfusion mice by inhibiting pyroptosis

**Fig. 4:**
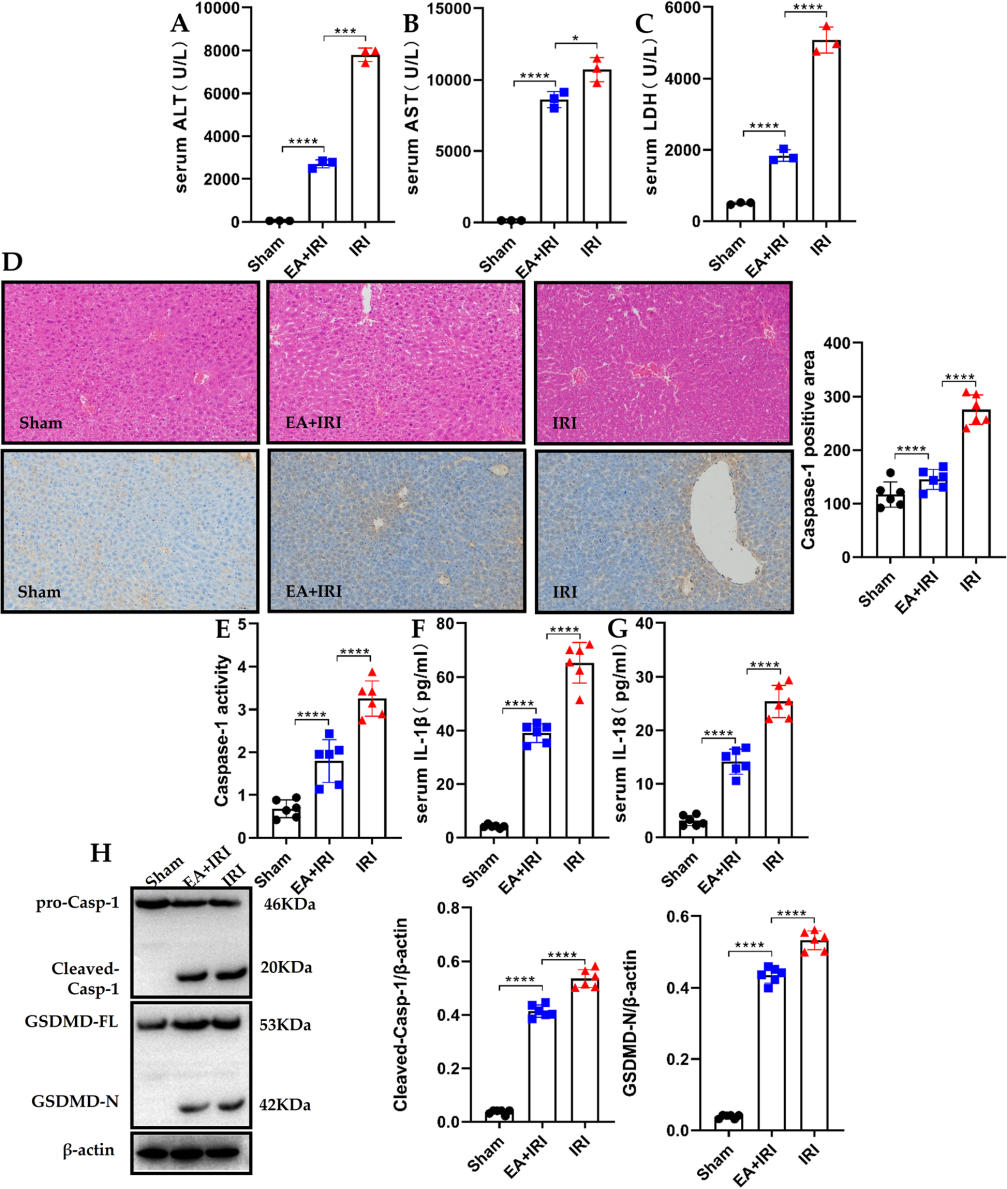
Ellagic acid reduces liver injury in hepatic ischemia–reperfusion mice by inhibiting pyroptosis. **A**, **B** and **C** The hepatocellular function in serum samples was evaluated by detecting ALT, AST, and LDH levels (*n* = 3). **D** Representative images of hematoxylin and eosin (HE) staining, and representative images of caspase-1 staining, the area of Caspase-1-positive cells in the livers were also determined (scale bar, 100 μm) (*n* = 6). **E** and **F** IL-1β and IL-18 were measured with ELISA in liver samples with Hepatic IRI, sham intervention and EA treatment mice (*n* = 6). **G** Caspase-1 activities were measured in liver tissues with Hepatic IRI, sham intervention and EA treatment mice (*n* = 6). **H** Examination of the proteolytic cleavage of Caspase-1 and GSDMD in mice liver specimens with hepatic IRI or sham intervention, using immunoblotting analysis. GSDMD-FL, full-length GSDMD; GSDMD-N, the N-terminal cleavage product of GSDMD (*n* = 6). All data are shown as the mean ± SEM. ****P < 0.0001, ***P < 0.001, **P < 0.01 and *P < 0.05

The essential role of Caspase-1-GSDMD-mediated pyroptosis in hepatic IRI prompted the investigation of whether the Caspase-1-GSDMD pathway can be a potential therapeutic target for hepatic IRI. Studies have shown that EA has a protective effect on the liver. This study demonstrates that early treatment with EA can reduce the ALT, AST and LDH levels in the blood and reduce liver injury (Fig. 4A, B, C). Further histopathological examination revealed significant features of severe centrilobular ballooning, congestion and lobular necrosis in the hepatic IRI model mice group. However, these findings were potently attenuated after the EA treatment (Fig. 4D), suggesting that EA could reverse hepatic ischemia- reperfusion injury.

Furthermore, immunohistochemistry (IHC) analysis of Caspase-1 specific markers for classic pyroptosis was carried out to assess hepatic pyroptosis. As shown in Fig. 4D, caspase-1 levels were significantly higher in the hepatic IRI model mice than in the sham group. Notably, EA administration robustly decreased the expression of Caspase-1 to levels comparable to the livers of sham mice. The serum levels of caspase-1 activity and mature IL-1β and IL-18 were up-regulated in the hepatic IRI model mice, whereas such an up-regulation was repressed by EA treatment (Fig. 4E, F, G). Correspondingly, after EA treatment, the expression of pyroptosis-related genes, proteolytic cleavage of both caspase-1 and GSDMD was inhibited in the hepatic IRI model mice, indicating that EA indeed suppressed pyroptosis associated with hepatic IRI *in vivo* (Fig. S1, Fig. 4H). The above data suggest that EA reduced liver injury in hepatic ischemia–reperfusion mice by inhibiting pyroptosis.

## Discussion

This study demonstrates that pyroptosis is involved in hepatic IRI, which is involved in increasing the expression of pyroptosis-related genes and promoting the expression of active Caspase-1, thereby cleaving GSDMD-N to cause pyroptosis. Importantly, we found that inhibition of pyroptosis by caspase-1 ablation or EA treatment potently attenuates liver congestion and lobular necrosis caused by hepatic IRI. These findings reveal a previously unidentified underlying mechanism of hepatic ischemia reperfusion injury, providing a potential therapeutic option to prevent hepatic IRI in pets.

Hepatic IRI is an inevitable complication in the pet's process of partial liver hepatectomy. It is an important cause of postoperative liver dysfunction and even liver failure. The mechanism of hepatic IRI has not been fully elucidated. In human medicine research, at present, it is considered that it may be the result of the joint action of many factors such as elevated free radicals, intracellular calcium overload, leukocyte activation, microvascular dysfunction and so on [23]. There are many forms of cell death involved in hepatic IRI, the main ones being apoptosis and pyroptosis [24]. Our results found hepatic IRI disrupted the liver structure and impaired liver function, evidenced by the large number of inflammatory factors were detected in the liver. Pyroptosis is a form of programmed cell death that is different from apoptosis and necrosis and is dependent on inflammatory caspase-1 and/or caspase-11. The activation of caspase-1 is mediated by inflammatory complexes such as NLRP3 [25]. Although pyroptosis was first described in 1992 and later reported in 2001 [26, 27], the potential application of inflammasome-mediated inflammatory caspase activation leading to lytic cell death remains unclear. GSDMD has been described as the executioner of pyroptosis [28–30]. In the absence of stimulation, full-length GSDMD remains intact, with the N-terminal and C-terminal regions interacting with each other. The oligomerized form GSDMD-N translocates to the plasma membrane and exhibits membrane-disrupting cytotoxicity in mammalian cells [31, 32]. The results of IHC and WB indicated that pyroptosis was involved in hepatic IRI and promoted the expression of active Caspase-1, thereby cleaving GSDMD-N and then perforating the cell membrane. Pyroptosis has been implicated in various diseases, such as nonalcoholic fatty liver, gastric cancer, and myocardial infarction [33, 34]. This study demonstrates that the expression of pyroptosis-related genes is up-regulated, and the activity of Caspase-1 is increased, with an apparent activation of caspase-1-GSDMD in mice liver tissues during hepatic IRI, suggesting the possible involvement of pyroptosis in hepatic IRI. Moreover, depletion of caspase-1 (using Caspase-1^−/−^ C57BL/6 mice) can reduce liver damage and inflammation caused by hepatic IRI. Pyroptosis can be induced by a canonical signaling pathway and a non-canonical signaling pathway [22, 35]. In this study, we found that the protein levels and the activity of caspase-1 were remarkably enhanced during hepatic IRI, suggesting the activation of the canonical pyroptosis signaling pathway (caspase-1-GSDMD pathway).

EA is a naturally occurring polyphenolic compound that is found in many fruits and walnuts [15, 36]. Studies have shown that EA protects endothelial cells from apoptosis induced by oxidized low-density lipoprotein by regulating the PI3K/Akt/eNOS pathway [37], and also has antibacterial and anti-inflammatory effects [38]. Recent studies have shown that EA has a protective effect on the liver [18]. This was also confirmed by our findings, revealing that EA protected the liver structure while reducing the levels of chemokines, thereby reducing the accumulation of inflammatory cells. Serum ALT and AST are widely used as markers of liver injury [19]. In this study, EA (60 mg/kg) was administered to mice for 14 consecutive days before surgery to study the effects of EA on ischemia–reperfusion. The results show that EA can significantly reduce the liver injury caused by ischemia–reperfusion, which is manifested in the decrease of ALT and AST levels. In addition, histological analysis showed that EA could significantly reduce liver damage. Because EA can protect hepatic IRI, and Caspase-1-GSDMD-mediated pyroptosis is involved in hepatic IRI, we are interested in whether EA can protect hepatic IRI by inhibiting pyroptosis. The results show that fewer caspase-1 expressions were detected in the livers of EA-intervened mice. The caspase-1 activity and mature IL-1β and IL-18 levels were up-regulated in hepatic IRI model mice, whereas the same was repressed by EA treatment. Correspondingly, after EA treatment, proteolytic cleavage of both caspase-1 and GSDMD was inhibited in the hepatic IRI model mice, indicating that EA indeed suppressed pyroptosis associated with hepatic IRI *in vivo*. This evidence indicates that EA can protect against hepatic IRI by inhibiting the molecular mechanism of the caspase-1-GSDMD pathway.

**Fig. 5:**
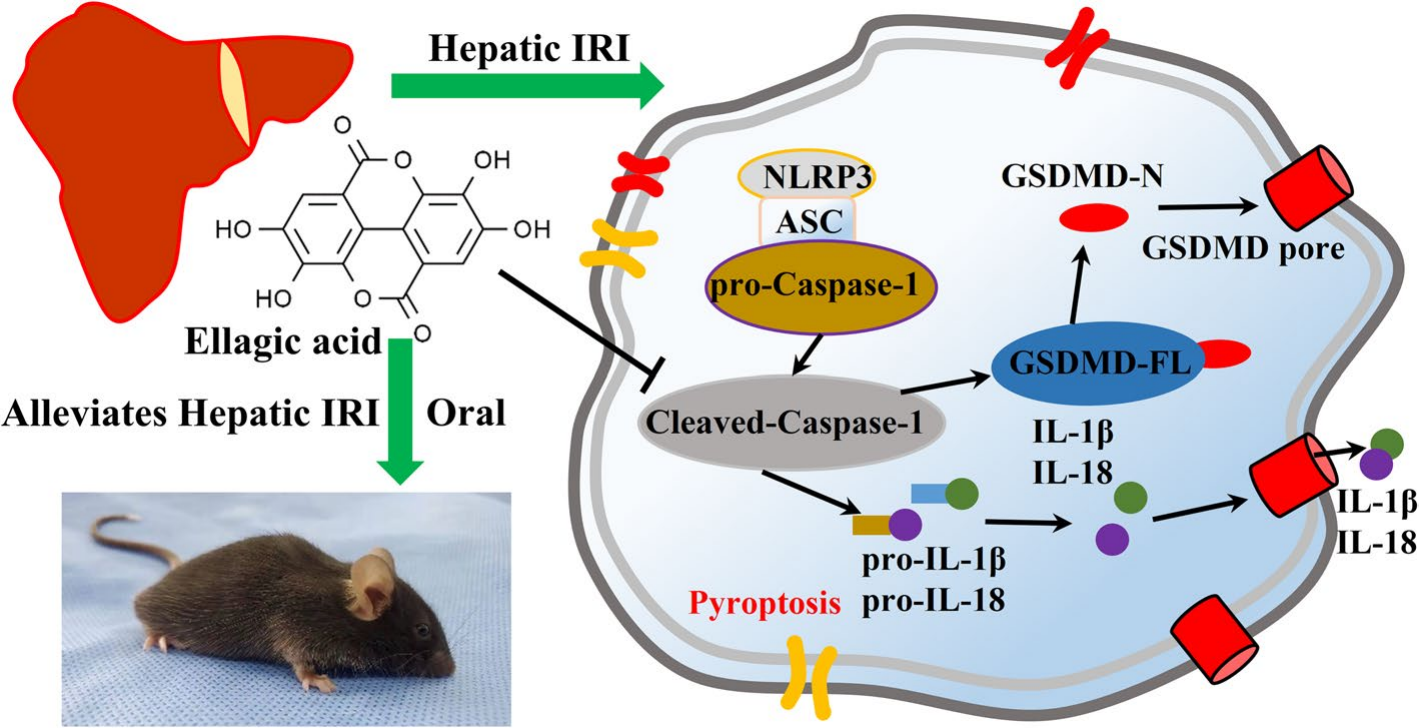
A schematic diagram illustrating that pyroptosis is involved in liver ischemia–reperfusion injury, and preventive supplementation of ellagic acid can regulate pyroptosis in hepatic ischemia–reperfusion injury

However, this study has some limitations. Although the involvement of pyroptosis in hepatic ischemia–reperfusion injury was demonstrated, its effects on hepatocytes and Kupffer cells were not explored. In addition, further experiments are required to investigate the types of cells in Fig. 5 The simplest interpretation of our data is that cells in the liver undergo pyroptosis in hepatic IRI, and EA protects the liver by inhibiting hepatic IRI-induced pyroptosis.

In summary, we found that EA ameliorated hepatic IRI-induced injury by inhibiting pyroptosis *in vivo* through the caspase-1-GSDMD pathway (The schematic diagram is shown in Fig. 5).

## Conclusions

This is the first study to inhibit Caspase-1-GSDMD-induced pyroptosis through dietary supplementation of EA, thereby protecting the liver from ischemia–reperfusion injury. The results demonstrated that the pyroptosis triggered by Caspase-1-GSDMD is involved in liver ischemia–reperfusion injury. In this study, prophylactic supplementation of EA for 2 weeks inhibited pyroptosis and improved liver function during hepatic ischemia–reperfusion injury. These new findings provide basic information that EA may regulate cell pyroptosis in liver ischemia–reperfusion injury, which may help reduce liver inflammation and injury in pets with ischemia–reperfusion. The results of this study provide basic information and suggest that prophylactic use of EA may be protective for pets with liver ischemia–reperfusion injury (such as after partial hepatectomy).

## Materials and methods

### Animals

Male wild-type and Caspase-1^−/−^ C57BL/6 mice (18-22 g) were purchased from Cyagen (Wuhan, China). The mice were kept under specific pathogen-free conditions, and the cages were located in a room with an alternating photoperiod of 12 h light and 12 h darkness, a temperature of 25 °C ± 2 °C, and ambient humidity (40–60%). The mice were then randomly assigned to experimental animal groups, and the experimental protocol was approved by the Animal Ethics Committee of Guizhou University.

### Hepatic IRI mice model and treatment

**Fig. 6:**
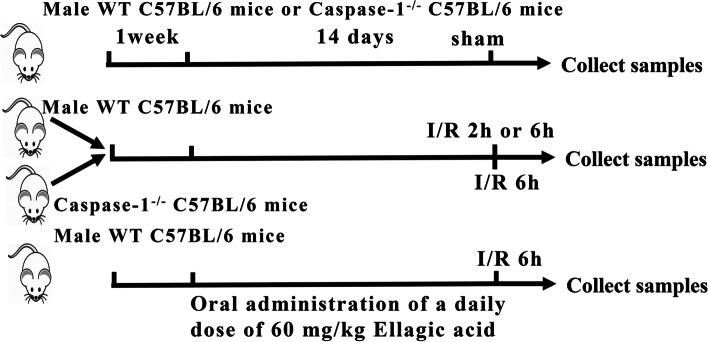
Summary of experiments design

As described in previous studies [39], a warm partial hepatic IRI model was used. In short, the control group (sham intervention, *n* ≥ 5) underwent laparotomy, but the hepatic portal veins were not obstructed. The mice in the hepatic IRI group were freed from the hepatic portal vein and blocked the blood supply to the mid lobe and left-hepatic lobe for an hour and a half, and the blood vessels were then opened for 2 h or 6 h. If the mice died before sampling, the sample was discarded. All operations were performed by the same operator, and the mice were allowed to fast for 14 h before the operation. Mice in the treatment group were subjected to oral administration of a daily dose of 60 mg/kg EA (Solarbio, Beijing, China) (add 0.6 g of EA to 100 mL normal saline.)for 14 days before performing surgery to model liver ischemia [20]. The experiment design is illustrated in Fig. 6.

### Biochemical measurement

Blood was collected from mouse eyeballs and centrifuged at 8,000 rpm for 10 min to obtain serum. Serum ALT, AST, and LDH levels were measured by microplate test kits (Jiancheng Bioengineering Institute, Nanjing, China). In short, the collected serum was reacted with a microplate, placed in an automatic biochemical analyzer (Mindray, BS-280), and the detection was performed at a wavelength of 340 nm.

### Histopathology and immunohistochemistry

Liver tissues were fixed with 4% paraformaldehyde overnight at 4 °C, followed by dehydration through an alcohol-xylene series, and finally embedded in paraffin. Livers ections (5 μm thickness) were cut (Leica2235, GER), dried at 37 °C, deparaffinized, rehydrated through a series of xylene-alcohol, rinsed with deionized water, and finally stained with H&E (Hematoxylin and Eosin).

With reference to UltraSensitive™ SP (Mice/Rabbit) IHC Kit (MXB, China), the slices were prepared in 0.01 M citrate buffer (pH 6.0) for antigen retrieval. The slices were immersed in 3% H_2_O_2_ for 10 min, and the non-immune goat serum was blocked for 10 min at room temperature. Subsequently, the solution was incubated with caspase-1 (Santa Cruz, 14F468) at 4 °C for about 12 h. The slices were washed 3 times with PBS (MXB, China) buffer and then placed with the secondary antibody at room temperature for 30 min. Subsequently, the sections were stained with DAB (MXB, China) for 5 min and then with hematoxylin for 10 min. The images were captured using the Olympus BX43F microscope (Tokyo, Japan). The caspase-1 positive areas were measured using ImageJ.

### Western blotting analysis

The western blotting experiment was carried out according to the protocol [40]. Briefly, liver tissues were collected according to the experimental protocol. Proteins were extracted from the liver tissue using a protein extraction kit (Thermo #78,510). The protein concentration was determined using the Bradford method, protein extracts were fractionated on a 12% sodium dodecyl sulfate–polyacrylamide gel and transferred to a nitrocellulose membrane, and the membrane was blocked with 5% nonfat milk for 2 h at room temperature. The membranes were then incubated with primary antibodies. Primary antibodies were used against mice caspase-1 (#ab179515) (Abcam, MA, USA), GSDMD (#ab209845) (Abcam, MA, USA), for these specific proteins, β-actin (Sigma-Aldrich St. Louis, MO, USA) was used as a loading control.

### RNA extraction and real‑time PCR

The total RNA was extracted from mice brain tissues with an RNAiso Plus kit (Takara, Dalian, China) according to the manufacturer’s instructions. One microgram of total RNA was reverse-transcribed to cDNA using the PrimeScript RT Master Mix kit (Takara, Dalian, China). Real-time PCR reactions were performed in triplicate using SYBR Premix Ex Taq II (Takara, Dalian, China) and analyzed with CFX96TM Real-time System (Bio-Rad, Hercules, CA, USA). The relative changes in mRNA were calculated using the ΔΔCt method and standardized to the housekeeping gene β-actin. The sequences of the primers used are provided in Table 1.

**Table 1:** Primers used for qPCR analysis

Name	Sequences(5’-3’)	Name	Sequences(5’-3’)
***β-actin***	CCTGCGGCATTCACGAAACTAC	***IL-18***	GGCTGCCATGTCAGAAGACT
XM_005887322.2	ACTCCTGCTTGCTGATCCACAAT	BC024384.1	CCTCGAACACAGGCTGTCTT
***NLRP3***	AACTGCAGCATCTCCTGGAC	***CXCL-1***	TTCACCTCAAGAACATCCAG
KF032621.1	ACACAATCCAGCAGACCAGG	NM_008176.3	TACTTGGGGACACCTTTTAG
***ASC***	AACCCAAGCAAGATGCGGAAG	***CXCL-2***	CTTCAAGAACATCCAGAGCT
AB059327.1	TTAGGGCCTGGAGGAGCAAG	NM_009140.2	ATGATTTTCTGAACCAGGGG
***Caspase-1***	GCCGTGGAGAGAAACAAGGA	***CXCL-10***	ACTGCATCCATATCGATGAC
BC008152.1	AAAAGTGAGCCCCTGACAGG	NM_021274.2	CTTTTTCATCGTGGCAATGA
***IL-1β***	AATGAAAGACGGCACACCCA		
NM_008361.4	GGAAGACAGGCTTGTGCTCT		

### ELISA assay

ELISA kits were used to detect mice serum levels of IL-18 and IL-1β (Jiancheng Bioengineering, Nanjing, China) according to the manufacturer’s protocols.

### Caspase-1 activity

Caspase-1 activity was detected in liver tissues. The activity was measured with a caspase-1 Assay Kit (Jiancheng Biotechnology, Nanjing, China) according to the manufacturer’s instructions.

### Statistical analysis

GraphPad Prism 8.0 software (GraphPad Software, San Diego, CA, USA) was used for statistical analysis. The results are expressed as mean ± SEM unless otherwise stated. The unpaired Student's t-test was used to compare the two groups, and the variances between the statistically compared groups were similar. A *P*-value of < 0.05 was considered significant.

## Supplementary Information

**Additional file 1: Fig. S1.** After EA treatment, the expression of pyroptosis-related genes was inhibited. **Fig. S2**, **Fig. S3** and **Fig. S4**: Uncropped western blot images.

## Data Availability

The datasets generated during and/or analyzed during the current study are available from the corresponding author on reasonable request. β-actin(XM_005887322.2, https://www.ncbi.nlm.nih.gov/search/all/?term=XM_005887322.2), NLRP3(KF032621.1, https://www.ncbi.nlm.nih.gov/search/all/?term=KF032621.1), ASC(AB059327.1, https://www.ncbi.nlm.nih.gov/search/all/?term=AB059327.1), Caspase-1(BC008152.1, https://www.ncbi.nlm.nih.gov/search/all/?term=BC008152.1), IL-1β(NM_008361.4, https://www.ncbi.nlm.nih.gov/search/all/?term=NM_008361.4), IL-18(BC024384.1, https://www.ncbi.nlm.nih.gov/search/all/?term=BC024384.1), CXCL-1(NM_008176.3, https://www.ncbi.nlm.nih.gov/search/all/?term=NM_008176.3), CXCL-2(NM_009140.2, https://www.ncbi.nlm.nih.gov/search/all/?term=NM_009140.20), CXCL-10(NM_021274.2, https://www.ncbi.nlm.nih.gov/search/all/?term=NM_021274.2). All data can be accessed in the National Center for Biotechnology Information.

## Abbreviations

EA: Ellagic acid
IRI: Ischemia–reperfusion injury
NLRP3: NLR family pyrin domain containing 3
ASC: Apoptosis associated speck like protein containing a CARD
caspase-1: Cysteinyl aspartate specific proteinase
GSDMD: Gasdermin D
IL-18: Interleukin 18
IL-1β: Interleukin-1β
