# Supplementary material for: Ellagic acid Alleviates hepatic ischemia–reperfusion injury in C57 mice via the Caspase-1-GSDMD pathway
Source: BMC Vet Res. 2022 Jun 18;18:229. doi: 10.1186/s12917-022-03326-0 (PMC9206301; doi:10.1186/s12917-022-03326-0)
Supplement: Supplementary file 1 — Additional file 1: Fig. S1. After EA treatment, the expression of pyroptosis-related genes was inhibited. Fig. S2, Fig. S3 and Fig. S4: Uncropped western blot images. [file 12917_2022_3326_MOESM1_ESM.pdf]

# Supplementary Materials

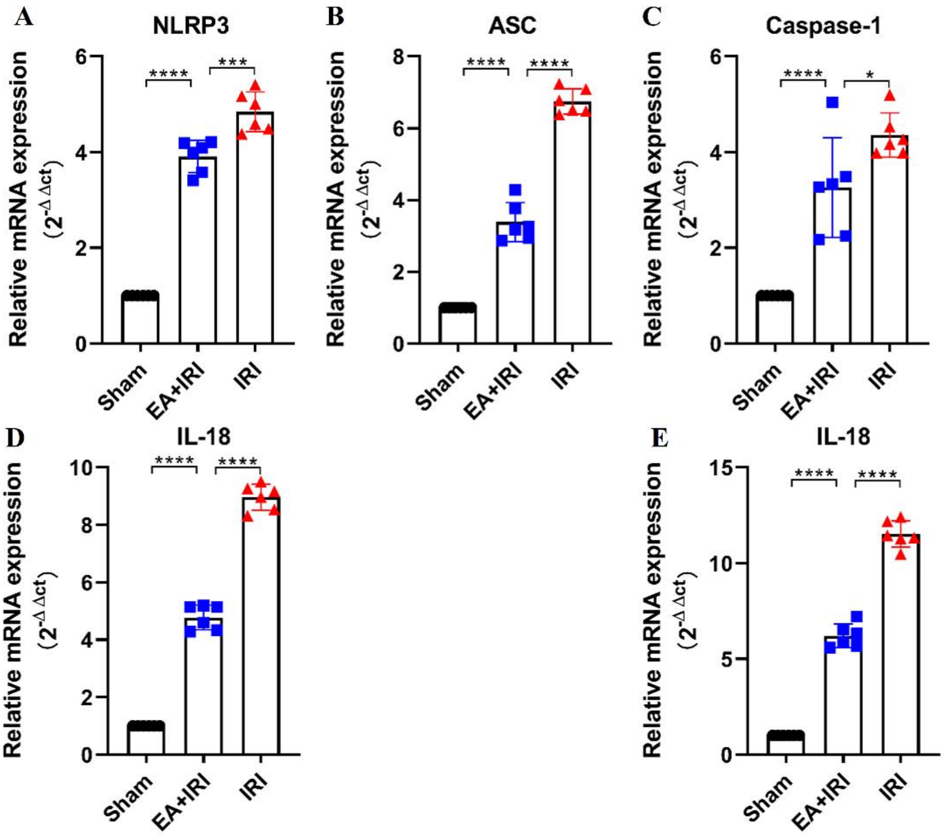


**Figure S1.** After EA treatment, the expression of pyroptosis-related genes was inhibited. (A-E) The expression levels of (A) NLRP3, (B) ASC, (C) Caspase-1, (D) IL-1β and (E) IL-18 are shown. All data are shown as the mean ± SEM. ****$P$< 0.0001， ***$P$< 0.001, **$P$ < 0.01 and *$P$< 0.05.

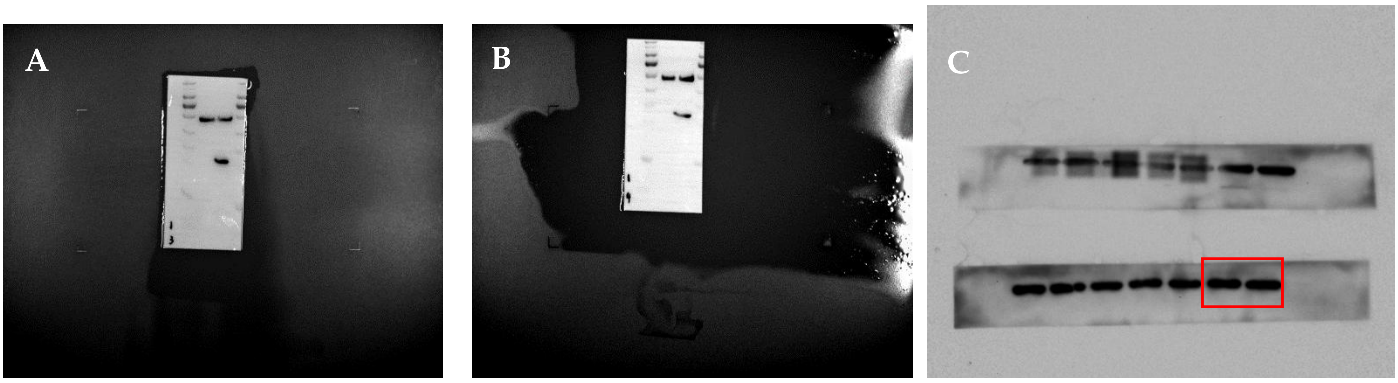


**Figure S2. Figure 3H uncropped western blot images.** (A) pro-Caspase-1 and Caspase-1 (Lane 1: Marker, Lane 2: sham group, Lane 3: IRI group); (B) GSDMD-FL and GSDMD-N (Lane 1: Marker, Lane 2: sham group, Lane 3: IRI group); (C) β-actin (Mark with red square).

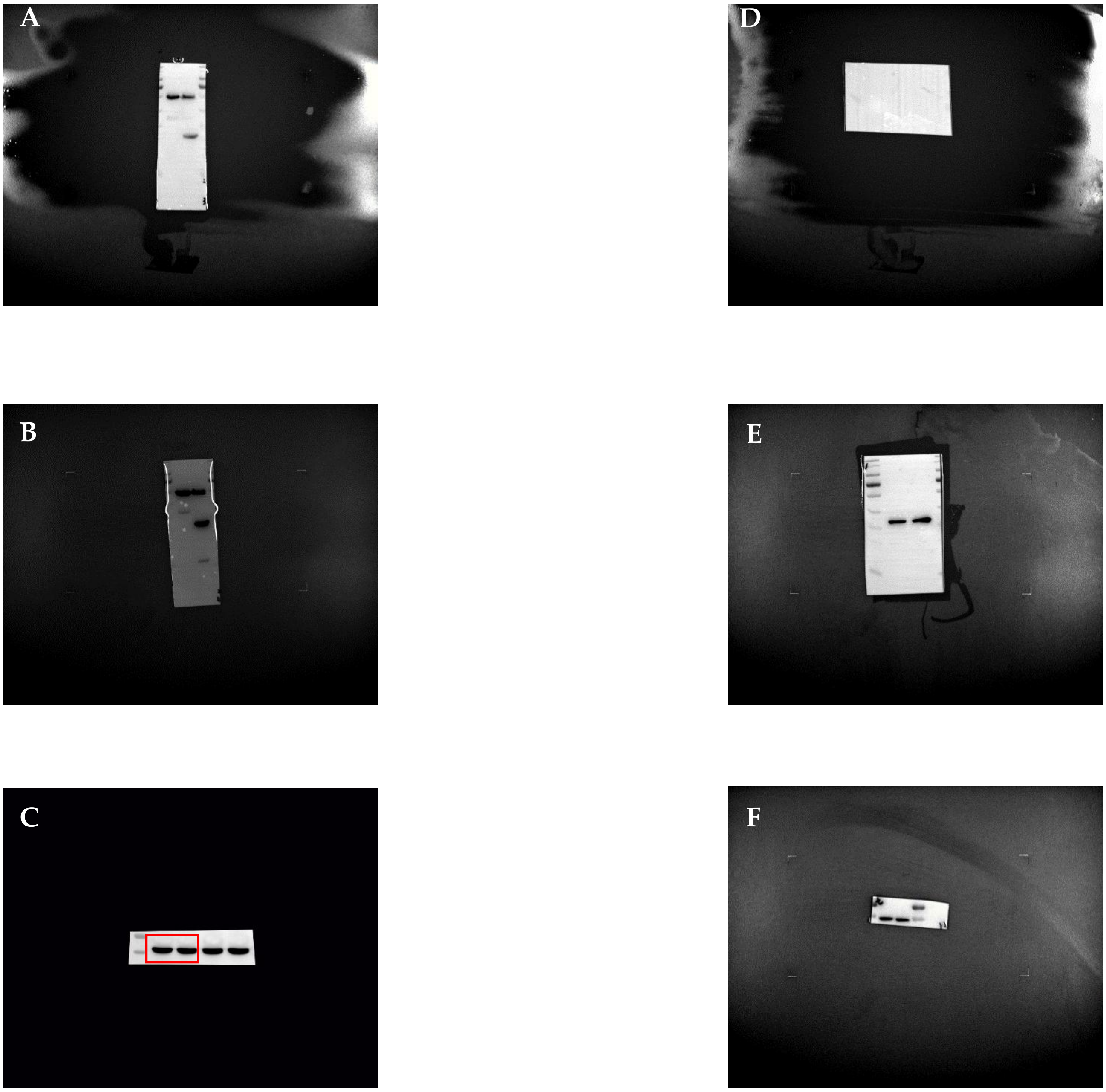


**Figure S3. Figure 4G uncropped western blot images.** (A) WT mice pro-Caspase-1 and Caspase-1 (Lane 1:Marker, Lane 2: sham-wt group, Lane 3: IRI-wt group); (B) WT mice GSDMD-FL and GSDMD-N (Lane 1: Marker, Lane 2: sham-wt group, Lane 3: IRI-wt group); (C) WT mice β-actin (Mark with red square, Lane 1: Marker, Lane 2: sham-wt group, Lane 3: IRI-wt group); (D) Caspase-1$^{-/-}$ mice pro-Caspase-1 and Caspase-1: (Lane1:sham-Caspase-1$^{-/-}$ group, Lane 2: IRI-Caspase-1$^{-/-}$ group); (E) Caspase-1$^{-/-}$ mice GSDMD-FL and GSDMD-N (Lane 1: Marker, Lane 2: sham-Caspase-1$^{-/-}$ group, Lane 3: IRI-Caspase-1$^{-/-}$ group,); (F) Caspase-1$^{-/-}$ mice β-actin (Lane 1: sham-Caspase-1$^{-/-}$ group, Lane 2: IRI-Caspase-1$^{-/-}$ group, Lane 3: Marker)

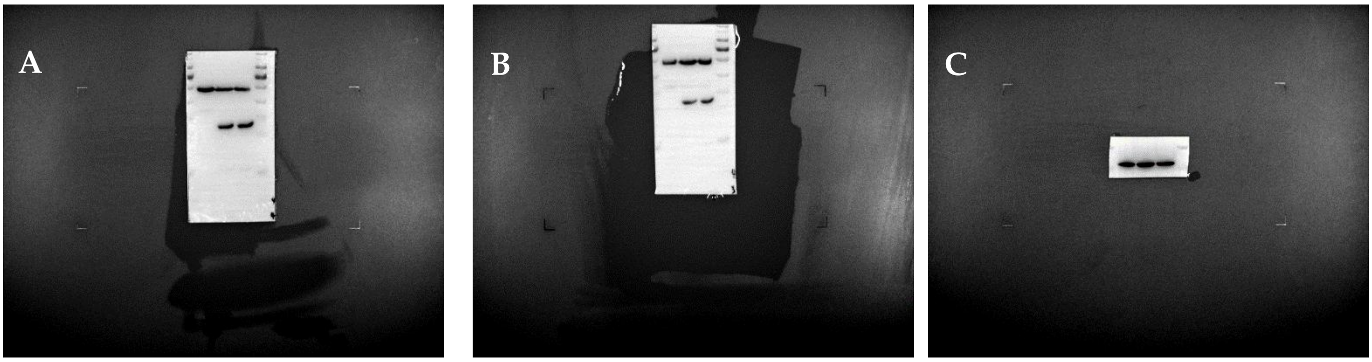


**Figure S4. Figure 5H uncropped western blot images.** (A) pro-Caspase-1 and Caspase-1 (Lane 1: Marker, Lane 2: sham group, Lane 3: EA+IRI group, Lane 4: IRI group ); (B) GSDMD-FL and GSDMD-N (Lane 1: Marker, Lane 2: sham group, Lane 3: EA+IRI group, Lane 4:IRI group ); (C) β-actin ( Lane 1: sham group, Lane 2: EA+IRI group, Lane 3: IRI group )
